# The Effects of N95 Masks on Sinonasal Symptoms and Opinions of Healthcare Workers in Singapore

**DOI:** 10.7759/cureus.88837

**Published:** 2025-07-27

**Authors:** Dhayan Parresh Timbadia, Shuhui Xu, Charn Tze Choong, Wei Yang Neville Teo

**Affiliations:** 1 Department of Otolaryngology - Head and Neck Surgery, Singapore General Hospital, Singapore, SGP; 2 Department of Otolaryngology - Head and Neck Surgery, SIngapore General Hospital, Singapore, SGP; 3 Department of Otolaryngology - Head and Neck Surgery, Sengkang General Hospital, Singapore, SGP

**Keywords:** blocked nose, healthcare provider, n95 respirators, rhinorrhea, sneezing

## Abstract

Background

The use of N95 masks is ubiquitous in the healthcare setting to protect against respiratory tract infections and even more so since the emergence of the SARS-CoV-2 virus (COVID-19) infection. The purpose of this study was to assess the effect of N95 masks on nasal symptoms.

Methods

We designed a questionnaire to survey nasal symptoms such as rhinorrhoea, obstruction, sneezing, and itching that may be experienced whilst wearing the different models of N95 masks. This was disseminated to staff from departments that had the highest usage of N95 masks, namely, accident and emergency, anaesthesia, and otolaryngology. The completed questionnaires underwent statistical analyses to identify the prevalence and effect of N95 masks on healthcare workers.

Results

A total of 232 survey responses were obtained. When analysing the responses across four major nasal complaints, 115 (49.6%) experienced nasal itching, 98 (42.2%) had rhinorrhoea, 79 (34.1%) developed a blocked nose, and 44 (19%) users experienced worse sneezing with mask wear. Nasal itch was also more commonly experienced than rhinorrhoea and nasal obstruction. Users with an existing history of allergic rhinitis were more likely to develop nasal symptoms with mask wear. There was no significant association between gender and nasal symptoms except for in the category of sneezing. There was also no significant association between worse symptoms and the three major mask models worn by users participating in our survey.

Conclusion

The use of N95 masks is associated with significant nasal complaints. This may be due to the formation of a microclimate within the mask’s dead space, which may alter the physiological environment of the nasal cavity. Additionally, factors such as a history of allergic rhinitis and exposure to polypropylene materials used in the mask's construction may further contribute to the development of nasal symptoms.

## Introduction

N95 masks are an essential component of personal protective equipment for healthcare workers to protect against respiratory tract infections. The importance of N95 masks is better appreciated during various respiratory outbreaks, such as the severe acute respiratory syndrome (SARS) outbreak in 2003, the H1N1 pandemic in 2009, and the most recent COVID-19 pandemic, which started in January 2020. While a meta-analysis did not find a significant difference between N95 respirators and surgical masks in preventing respiratory viral infections [1], there is evidence favouring the use of N95 masks during aerosol-generating procedures or when a patient’s COVID-19 status is positive or unknown [2]. As such, many healthcare workers were required to wear N95 masks for prolonged periods.

Multiple studies have looked at the adverse effects of N95 masks on various body systems and have found that usage of N95 masks can alter physiological function [3], impair cardiopulmonary function [4], and cause adverse skin reactions [5,6]. Over the course of the COVID-19 pandemic, there have also been several studies published looking at the effects of both surgical masks and N95 respirators on sinonasal complaints [7,8].

The nasal cavity consists of a large variety of structures and processes, which come together to maintain the internal environment of the upper respiratory tract. Factors such as airflow, temperature, pH, and humidity are important in maintaining nasal physiology [9,10]. During the use of N95 masks, breathing occurs through the pores and filtering devices. N95 masks are known to alter these variables, which may lead to alterations in natural physiology [11]. The study by Zhu et al. showed that nasal resistance increased after prolonged N95 mask wear [12]. A more recent study also showed that N95 mask use leads to prolongation of nasal mucociliary clearance [8].

At the time of study initiation, there was no published literature describing the effects of N95 mask use on nasal complaints. While one would expect nasal obstruction to be associated with N95 mask wearing, there were also reports from fellow colleagues who suffered from other nasal symptoms, such as rhinorrhoea, sneezing, and nasal itch, significantly. Some also switched to different models of N95 masks in an attempt to relieve their nasal symptoms. As such, this study was conducted to investigate the prevalence of nasal symptoms among healthcare workers using N95 masks in Singapore.

## Materials and methods

This study was conducted across three hospitals within the SingHealth Duke-NUS Academic Medical Centre from 15th September 2021 to 31st May 2022. This included Singapore General Hospital, Changi General Hospital, and Sengkang General Hospital. An anonymous online questionnaire was sent out to doctors and nurses from the departments of accident and emergency, anaesthesiology, and otorhinolaryngology - head and neck surgery, as healthcare workers from these departments were wearing N95 masks on a daily basis and were noted to have the highest usage of N95 mask wear during the time of the survey. The questionnaire was based on nasal symptoms that participants may commonly experience. No testing of the survey was done prior to the study. The final questionnaire included questions regarding symptoms of nasal obstruction, rhinorrhoea, itchy nose, and increased sneezing when wearing N95 masks. We also went on to further quantify the time taken for these symptoms to manifest after donning N95 masks, the duration for which these symptoms last, and the duration for which these symptoms persist even after the removal of N95 masks. We also asked participants subjectively which brands of masks caused the most and least sinonasal symptoms. The three most commonly used masks at that time across these three hospitals were the 3M^TM^ Medical Respirator 1860/1860S N95 (3M, St. Paul, MN), the 3M^TM^ Aura^TM^ Health Care Particulate Respirator and Surgical Mask 1870+ N95, and the Air+ Protect Mask (AIR⁺, Singapore).

The 3M^TM^ Medical Respirator 1860/1860S N95 mask had 99% bacterial filtration efficiency (BFE) according to ASTM F2101, with a collapse-resistant cup shape design, braided headbands, cushioning nose foam, and lightweight construction for comfortable wear. The 3M^TM^ Aura^TM^ Health Care Particulate Respirator and Surgical Mask 1870+ similarly was a 99% BFE mask, according to ASTM F2101. It, however, had a sculpted nose panel that follows the contours of your nose, allowing more room for eyewear, an embossed top panel that was designed to help reduce the fogging of eyewear from warm, moist exhaled air, and an innovative chin tab designed for ease of positioning, donning, and adjustment. Lastly, the Air+ Protect Mask was a filtering facepiece (FFP) 2 mask, certified under the European standard EN 149, with at least 94% of particles, including oil-based particles, making it versatile for various industrial settings. It had a plush nose cushion and an adjustable head strap for better facial seal and flexibility.

Statistical analysis was performed using SPSS version 23 (IBM Corp., Armonk, NY). The Pearson chi-square test and the Cochrane Q test were used for further analysis between variables and nasal symptoms. The significance level was calibrated at p < 0.05.

## Results

A total of 232 healthcare workers completed the online questionnaire, which consisted of 167 (72%) female participants and 65 (28%) male participants. The survey was carried out by healthcare workers across three major departments. A total of 78 participants were from the emergency department, while there were 68 and 86 participants from the anaesthesia and the otolaryngology department, respectively. The majority of our participants fell in the age group of 31-50 years old (54.7%), with the rest being 18-30 years old (24.6%) and 51-70 years old (20.7%). Only a very small proportion of our participants were smokers (3%). Most participants did not have any underlying nasal conditions (62.1%). Of the participants, 34% reported having a history of allergic rhinitis, while 6.9% of participants reported having a history of asthma. The most commonly donned mask was the 3M^TM^ Medical Respirator 1860/1860S N95 (41.4%), followed by 3M^TM^ Aura^TM^ Health Care Particulate Respirator and Surgical Mask 1870+ N95 (40.1%) and Air+ Protect Mask (15.1%) (Table 1).

**Table 1:** Descriptive statistics of our surveyed population. ^* ^3M^TM^ Medical Respirator 1860/1860S N95. ^†^ 3M^TM^ Aura^TM^ Health Care Particulate Respirator and Surgical Mask 1870+ N95. ^‡^ Air+ Protect Mask (15.1%).

		Frequency	Percentage (%)
Gender	Female	167	72
	Male	65	28
Smoker	Yes	7	3
	No	232	97
Age (years)	18-30	57	24.6
	31-50	127	54.7
	51-70	48	20.7
Underlying respiratory or nasal condition	Allergic rhinitis	79	34
	Asthma	16	6.9
	Nil	144	62.1
	Others	5	2
Department	Emergency department	78	33.6
	Anaesthesia	68	29.3
	Otolaryngology	86	37.1
Common N95 masks used	1860/1860S *	96	41.4
	1870+ ^†^	93	40.1
	Air+ ^‡^	35	15.1
	Others	8	0.4

Nasal obstruction

A total of 79 participants (34.1%) developed nasal obstruction while wearing N95 masks. Most users reported experiencing nasal obstruction after more than one hour of continued mask wear (55.7%). In a small number of participants, the symptoms of nasal obstruction started immediately (8.9%), while in 35.4% of participants, this started after a few minutes. Most participants continued feeling this sensation intermittently (49.4%) or constantly (40.5%) during mask wear. Only 22.8% of participants reported their symptoms resolving immediately after the removal of their masks, while in 48.1% symptoms resolved after a few minutes, and in 29.1% of participants, symptoms persisted for an hour longer. In the majority of our participants (77.2%), these symptoms led to mouth breathing. Among these users, 13.1% of users reported that this resulted in a sore throat most of the time, and 47.5% reported sometimes having a sore throat (Table 2).

**Table 2:** Nasal symptoms with N95 mask use. Table showing the presence of four major sinonasal symptoms (nasal obstruction, rhinorrhoea, nasal itch, and sneezing) in the presence of N95 mask wear. Further analysis of the time taken for the onset, duration, and persistence of symptoms after removal of the mask is also shown. The Pearson chi-square test was used for analysis between variables. The significance level was calibrated at P < 0.05. The Cochran's Q test and a pair-wise comparison using Dunn’s test were used.

		Nasal obstruction, n (%)	Rhinorrhoea, n (%)	Nasal itch, n (%)	Sneezing, n (%)	P-value	Chi-square value
Presence of symptoms while wearing an N95, worse than when not wearing one?	Yes	79 (34.1)	98 (42.2)	115 (49.6)	44 (19.0)	<0.001	51.9
	No	153 (65.9)	134 (57.8)	117 (50.4)	188 (81.0)		
Onset of symptoms	Immediately after wearing the mask	7 (8.9)	7 (7.1)	11 (9.6)	6 (13.6)		
	After a few minutes of mask wear	28 (35.4)	34 (34.7)	40 (34.8)	18 (40.9)		
	After >1 hour of mask wear	44 (55.7)	57 (58.2)	64 (55.7)	20 (45.5)		
Duration of symptoms while wearing N95	For a few minutes	1 (1.3)	7 (7.1)	13 (11.3)	8 (18.2)		
	More than a few minutes; resolves while wearing the mask	7 (8.9)	10 (10.2)	17 (14.8)	8 (18.2)		
	Intermittently, while wearing the mask	39 (49.4)	63 (64.3)	63 (54.8)	21 (47.7)		
	Constantly, while wearing the mask	32 (40.5)	18 (18.4)	22 (19.1)	7 (15.9)		
Persistence of symptoms after removal of N95	Stops immediately after the removal of the mask	18 (22.8)	17 (17.3)	37 (32.2)	16 (36.4)		
	A few minutes after the removal of the mask	38 (48.1)	51 (52.0)	56 (48.7)	15 (34.1)		
	An hour or longer after the removal of the mask	23 (29.1)	30 (30.6)	22 (19.1)	13 (29.5)		
Time taken for symptoms to resolve after removal of the mask	Symptoms resolve within 1 hour of the removal of the mask	56 (70.9)	68 (69.4)	93 (80.9)	31 (70.5)	0.634	4.56
	Symptoms persist for an hour or longer after removal of the mask	23 (29.1)	30 (30.6)	22 (19.1)	13 (29.5)		
Does the nasal obstruction lead to mouth breathing?	Yes	61 (77.2)	-	-	-		
	No	18 (22.8)	-	-	-		
Does mouth breathing result in a sore throat?	Yes	8 (13.1)	-	-	-		
	No	24 (39.3)	-	-	-		
	Sometimes	29 (47.5)	-	-	-		

Rhinorrhoea

Of all participants, 42.2% reported experiencing rhinorrhoea when wearing an N95 mask, most of whom started having symptoms only after one hour or longer of mask wear (58.2%). In 34.7% of respondents, symptoms started after a few minutes, while the rest (7.1%) had the onset of rhinorrhoea immediately after donning their masks. A majority of participants with rhinorrhoea reported having continued symptoms intermittently (64.3%) or constantly (18.4%) while wearing the mask. A majority of respondents (52%) reported resolution of rhinorrhoea after a few minutes of removal of the mask (Table 2).

Nasal itch

Almost half of all participants (49.6%) experienced an itchy nose while wearing an N95 mask. Most participants started experiencing this after one hour or longer of mask wear (55.7%). Symptoms tended to last throughout mask wear, with 54.8% of participants reporting symptoms lasting intermittently during mask wear and 19.1% experiencing itchiness constantly. In most participants, symptoms resolved immediately (32.2%) or within a few minutes (48.7%) after removal of the N95 mask (Table 2).

Sneezing

Sneezing was the least prevalent symptom among participants, with only 19% of all participants experiencing it while wearing N95 masks. Out of 44 participants, six (13.6%) experienced sneezing immediately after wearing the mask, 18 (40.9%) experienced it after a few minutes, and 20 (45.5%) experienced it after one hour or longer of mask wear. Most participants experienced sneezing intermittently throughout mask wear (47.7%). Duration of symptoms after removal of the mask was fairly distributed, with 36.4% claiming symptoms resolved immediately after removal of the mask, 34.1% reporting symptoms improving within a few minutes, and 29.5% of participants reporting symptoms resolving after an hour or longer (Table 2).

Further analysis using the Cochran's Q test [13] indicated a significant difference among the four proportions of sinonasal symptoms (X3 (3) = 65.7, P < 0.001). A pair-wise comparison using Dunn’s test [14] showed that nasal itch was more frequently experienced than both rhinorrhoea (P = 0.001) and nasal obstruction (P < 0.001) (Table 2).

We also further subdivided the time taken for symptoms to resolve into more or less than one hour. However, further analysis showed there was no significant difference in the duration taken for symptoms to resolve across the four sinonasal symptoms (P = 0.634) (Table 2).

Among the 232 respondents, 50 (21.6%) did not experience any sinonasal symptoms, hence did not require changing the model of the mask. Of those who experienced persistent nasal symptoms, 118 (64.8%) chose not to change the model of mask worn, while 64 (35.2%) tried switching to another model of mask. Amongst the users who decided to change their masks, nasal itch (64.6%) and sneezing (63.1%) were the most commonly experienced symptoms. A total of 56.3% of users claimed that the 3M^TM^ Aura^TM^ Health Care Particulate Respirator and Surgical Mask 1870+ N95 mask caused the least symptoms, while 65.6% of users claimed the 3M^TM^ Medical Respirator 1860/1860S N95 mask caused the most symptoms (Table 3).

**Table 3:** Association of brands of N95 masks and symptoms. Table showing users' perspective on which brand of N95 causes the most and least symptoms, and the number of users who attempted to switch masks due to sinonasal symptoms.

		Frequency	Percentage (%)
Users who tried switching to another model of mask given persistent nasal symptoms	No change required (Nil nasal symptoms)	50	21.6
	No	118	50.9
	Yes	64	27.6
Amongst participants who tried switching to another model of mask…			
Which model of mask causes the least symptoms?	1860/1860s	6	9.4
	1870+	36	56.3
	Air+	16	25
	Others	6	9.4
Which model of mask causes the most symptoms?	1860/1860s	42	65.6
	1870+	8	12.5
	Air+	8	12.5
	Others	6	9.6

Upon further analysis, participants with a self-reported history of allergic rhinitis were more likely to experience symptoms of rhinorrhoea (P = 0.023), itchy nose (P = 0.009), and sneezing (P < 0.001) during mask wear. However, users were not more likely to experience nasal obstruction (P = 0.111) (Table 4).

**Table 4:** Association of symptoms with a history of allergic rhinitis. Table showing the association of symptoms while wearing N95 masks in people with and without a history of allergic rhinitis. Chi-square test was used for further analysis between variables. The significance level was calibrated at p < 0.05.

	Symptoms worsen with mask wear			P-value	Chi-square value
	Yes (%)		No (%)		
The feeling of a blocked nose worsens with N95 mask wear					
History of allergic rhinitis	32 (41)	46 (59)		0.111	2.54
Nil history of allergic rhinitis	47 (30.5)	107 (69.5)			
The feeling of rhinitis worsens with N95 mask wear					
History of allergic rhinitis	41 (52.6)	37 (47.4)		0.023	5.13
Nil history of allergic rhinitis	57 (37)	97 (63)			
The feeling of an itchy nose worsens with N95 mask wear					
History of allergic rhinitis	48 (61.5)	30 (38.5)		0.00	6.73
Nil history of allergic rhinitis	67 (43.5)	87 (56.6)			
Experiencing sneezing worsens with N95 mask wear					
History of allergic rhinitis	25 (32.1)		53 (67.9)	P-value <0.001	13.09
Nil history of allergic rhinitis	19 (12.3)		135 (87.7)		

When the prevalence of nasal symptoms was compared across gender (Table 5), there were no significant differences between males and females for nasal obstruction (P = 1.000), rhinorrhoea (P = 0.767), and itchy nose (P = 0.307).

**Table 5:** Association of symptoms with gender. The chi-square test was used for further analysis between variables and nasal symptoms. The significance level was calibrated at p < 0.05.

	Symptoms worsen with mask wear			P-value	Chi-square value
	Yes (%)		No (%)		
Nasal obstruction					
Female	57 (34.1)	110 (65.9)		1.000	0.002
Male	22 (33.8)	43 (66.2)			
Rhinorrhoea					
Female	72 (43.1)	95 (56.9)			0.186
Male	26 (40)	39 (60)		0.767	
Itchy nose					
Female	79 (47.3)	88 (52.7)			1.22
Male	36 (55.4)	29 (44.6)		0.307	
Sneezing					
Female	25 (15)		142 (85)		6.19
Male	19 (29.2)		46 (70.8)	P-value = 0.016	

However, females were more likely to experience sneezing during mask wear (P = 0.016). Similarly, there was no significant difference across mask brands in the categories of nasal obstruction (P = 0.658), rhinorrhoea (P = 0.254), and sneezing (P = 0.935) (Table 6).

**Table 6:** Association of symptoms with mask brands. The chi-square test of independence was used for further analysis between variables and nasal symptoms. The significance level was calibrated at p < 0.05.

Symptoms worsen with mask wear			P-value	Chi-square value
	Yes (%)	No (%)		
Nasal obstruction			0.658	1.61
1860s/1860	30 (31.3)	66 (68.8)		
1870	31 (33.3)	62 (66.7)		
Air+	15 (42.9)	20 (57.1)		
Others	3 (37.5)	5 (62.5)		
Rhinorrhoea			0.254	4.07
1860s/1860	36 (37.5)	60 (62.5)		
1870	41 (44.1)	52 (55.9)		
Air+	19 (54.3)	16 (45.7)		
Others	2 (25)	6 (75)		
Itchy nose			0.201	4.63
1860s/1860	54 (56.3)	42 (43.8)		
1870	47 (50.5)	46 (49.5)		
Air+	25 (71.4)	10 (28.6)		
Others	4 (50)	4 (50)		
Sneezing			0.935	0.426
1860s/1860	18 (18.8)	78 (81.3)		
1870	19 (20.4)	74 (79.6)		
Air+	6 (17.1)	29 (82.9)		
Others	1 (12.5)	7 (87.5)		

## Discussion

Hospitals around the world continue to recommend and enforce the use of face masks and respirators during the COVID-19 Pandemic. The World Health Organization (WHO) recommends the use of respirators to protect health workers who provide care to COVID-19 patients in settings and areas where aerosol-generating procedures are carried out. N95 masks are also recommended for health professionals who are taking care of suspected or confirmed COVID-19 patients [15]. Most N95 masks consist of four layers of materials to aid in the filtration process. The most commonly used materials are polymeric materials such as polypropylene and polyethylene [16]. These masks help with filtration through electrostatic attraction and impaction by fibrous medium [17].

In this study of 232 participants, our findings suggest that wearing masks can lead to various nasal symptoms. The use of N95 masks can lead to an increase in the effort of breathing by a significant percentage. Previous studies have also shown that there is a known objective impairment of the nasal airflow with the use of such masks [18,19]. The majority of users experiencing the feeling of nasal obstruction continued having this either intermittently or constantly throughout mask wear (49.4% and 40.5%, respectively). In turn, this would also lead to many users opting to mouth breathe instead, which consequently can lead to sore throats and other complications [20,21]. Previous studies have also noted that mouth breathing can lead to higher amounts of water loss as compared to nose breathing [22]. This may contribute to the worsening feeling of throat discomfort and sore throat.

Studies have shown that N95 respirators result in a difference in temperature between the external environment and the microclimate environment within the dead space of the mask [3]. This can lead to a reduction in heat transfer in the human nasal cavity [11]. It is well known that mask wear leads to an alteration of the physiology of the nasal cavity and upper respiratory tract. Changes in the microclimate within the mask, such as the temperature, humidity, and airflow, can all impair the function of the structures within the nasal cavity. A recent study showed that there was a significant prolongation in the mucociliary clearance time amongst users who wore an N95 mask [7]. An impaired mucociliary function can lead to nasal complaints such as nasal obstruction and rhinorrhoea. In our study, 42.2% of participants complained of rhinorrhoea, and 34.1% of users complained about nasal obstruction. Comparatively, another study that evaluated nasal symptoms in prolonged face mask usage reported 30.4% of participants with nasal congestion or stuffiness, and 22.9% of participants who experienced any nasal obstruction or blockage [23].

In our study, close to half of all participants (49.6%) reported the feeling of having an itchy nose. Several previous studies have also shown that N95 masks can lead to a multitude of skin conditions. This may be due to a form of contact dermatitis between the materials used and the skin [5,6]. Other similar studies on face masks yielded similar results with an increased prevalence of allergy symptoms such as discomfort and itchiness [24]. Similarly, nasal itch was also more frequently experienced than both rhinorrhoea and nasal obstruction. The high prevalence of nasal itch may be explained due to the dual cause of the symptom. Itch may be both pruritoceptive [25,26] and nociceptive, and it is plausible that both mechanisms exist [27].

More interestingly, it was noted that patients who had a pre-existing history of allergic rhinitis were more likely to experience symptoms of rhinorrhoea, itchy nose, and sneezing during mask wear. Klimek et al. explored the effect of possible irritant dermatitis due to the usage of filtering facepiece (FFP) masks. Irritant rhinitis (IR) is defined as an inflammatory response of the nasal mucosa due to non-allergic stimuli. Participants who were wearing FFP masks for a minimum of two hours were noted to report similar nasal symptoms. Endoscopic findings of irritation and oedema with mucosal swelling and watery secretions were also noted. However, eosinophilic cationic protein (ECP) and total immunoglobulin E (IgE) levels were not registered to be raised [28]. As mentioned above, previous studies and reports have shown a possibility of allergic contact dermatitis and urticaria due to polypropylene [29-31]. Despite this, there is only a limited amount of literature on the predisposition of patients with a prior history of allergic rhinitis and nasal symptoms while wearing N95 masks.

Despite there not being any significant association between mask brands and the prevalence of nasal symptoms worsening with mask wear, more users subjectively felt that the 3M^TM^ Medical Respirator 1860/1860S N95 caused the worst symptoms (65.6%). Studies have shown that mask shape, style, and structural features may have a significant impact on the subjective feeling of comfort as well as the microclimate within the mask [32,33]. These results are significant, as discomfort with N95 mask use may lead to improper wearing of the masks or impact work performance. Further studies need to be done to evaluate the impact of such factors on nasal symptoms amongst N95 mask users.

Limitations of our study include the subjective nature of an online questionnaire, as there may be differences in the perceived impact of these symptoms. This may lead to variability and imprecision in the reporting of these symptoms. As the survey was voluntary, there may be non-response bias, with responders more likely to have experienced nasal symptoms during N95 mask use. Furthermore, the data were collected through a self-reported questionnaire, which is inherently susceptible to subjectivity and recall bias. The study lacks objective markers to quantify and measure the severity of nasal symptoms, such as corroboration of endoscopic findings or imaging investigation. Future studies can aim to focus on more objective markers, which may prove the increased prevalence of sinonasal symptoms in N95 mask users. Further research is also needed to investigate the interplay between polypropylenes and allergic rhinitis.

## Conclusions

Nasal itch is the most commonly reported symptom associated with N95 mask use, followed by rhinorrhoea, nasal obstruction, and, finally, sneezing. Most of the symptoms start after one hour of continued mask wear and persist as long as the masks are being worn, and resolve within one hour of mask removal. Patients with allergic rhinitis were significantly more likely to complain of sneezing, nasal itch, and rhinorrhoea. Certain N95 mask models elicited less pronounced nasal symptoms subjectively. Nasal discomfort with N95 mask wearing can lead to inefficient use and reduced compliance with mask usage, which may lead to reduced levels of protection against respiratory viruses. Further studies need to be done to find out the underlying cause of these symptoms to efficiently tackle them and improve comfort and confidence during the usage of such masks.

## Appendices

Questionnaire

1. Current hospital you are working at?

a. Singapore General Hospital

b. Changi General Hospital

c. Sengkang General Hospital

2. Current department you are working at?

a. Department of Otorhinolaryngology

b. Department of Accident and Emergency 

3. Gender?

a. Male

b. Female

4. Age group?

a. 18-30 years old

b. 31-50 years old

c. 51-70 years old

5. Model of N95 mask used most often?

a. 3M^TM^ Medical Respirator 1860/1860S N95 mask

b. 3M^TM^ Aura^TM^ Health Care Particulate Respirator and Surgical Mask 1870+ N95 mask

c. Air+ Protect Mask

d. Other

6. Are you currently a smoker?

a. Yes

b. No

7. Do you have any of these respiratory or nasal conditions?

a. Allergic rhinitis

b. Asthma

c. Others

8. Sinonasal symptoms

(i) Blocked nose

• Do you experience the feeling of having a blocked nose while wearing an N95 mask that is worse than when not wearing one?

a. Yes

b. No

• When does this start?

a. Immediately after I start wearing the mask

b. After a few minutes of mask wear

c. After one hour or longer of continued mask wear

• How long does this last for?

a. For a few minutes

b. Longer than a few minutes, but resolves while wearing the mask

c. Intermittently, so long as I am wearing the mask

d. Constantly, while wearing the mask

• How long does this persist after taking off the mask?

a. Stops immediately after removal of the mask

b. A few minutes after the removal of the mask

c. An hour or longer after the removal of the mask

• Does the nasal obstruction result in mouth breathing during mask wear?

a. Yes

b. No

• Does mouth breathing result in a sore throat?

a. Yes

b. No

(ii) Runny nose

• Do you experience the feeling of having a runny nose while wearing an N95 mask that is worse than when not wearing one?

a. Yes

b. No

• When does this start?

a. Immediately after I start wearing the mask

b. After a few minutes of mask wear

c. After one hour or longer of continued mask wear

• How long does this last for?

a. For a few minutes

b. Longer than a few minutes, but resolves while wearing the mask

c. Intermittently, so long as I am wearing the mask

d. Constantly, while wearing the mask

• How long does this persist after taking off the mask?

a. Stops immediately after removal of the mask

b. A few minutes after the removal of the mask

c. An hour or longer after the removal of the mask

(iii) Itchy nose

• Do you experience the feeling of having an itchy nose while wearing an N95 mask that is worse than when not wearing one?

a. Yes

b. No

• When does this start?

a. Immediately after I start wearing the mask

b. After a few minutes of mask wear

c. After one hour or longer of continued mask wear

• How long does this last for?

a. For a few minutes

b. Longer than a few minutes, but resolves while wearing the mask

c. Intermittently, so long as I am wearing the mask

d. Constantly, while wearing the mask

• How long does this persist after taking off the mask?

a. Stops immediately after removal of the mask

b. A few minutes after the removal of the mask

c. An hour or longer after the removal of the mask

(iv) Sneezing

• Do you experience sneezing while wearing an N95 mask that is worse than when not wearing one?

a. Yes

b. No

• When does this start?

a. Immediately after I start wearing the mask

b. After a few minutes of mask wear

c. After one hour or longer of continued mask wear

• How long does this last for?

a. For a few minutes

b. Longer than a few minutes, but resolves while wearing the mask

c. Intermittently, so long as I am wearing the mask

d. Constantly, while wearing the mask

• How long does this persist after taking off the mask?

a. Stops immediately after removal of the mask

b. A few minutes after the removal of the mask

c. An hour or longer after the removal of the mask

9. Have you tried switching to another model of N95 mask in view of your nasal symptoms?

a. Yes

b. No

10. If yes, which model of N95 mask causes the LEAST amount of symptoms?

a. 3M^TM^ Medical Respirator 1860/1860S N95 mask

b. 3M^TM^ Aura^TM^ Health Care Particulate Respirator and Surgical Mask 1870+ N95 mask

c. Air+ Protect Mask

d. Other

11. Which model of N95 mask causes the MOST amount of symptoms?

a. 3M^TM^ Medical Respirator 1860/1860S N95 mask

b. 3M^TM^ Aura^TM^ Health Care Particulate Respirator and Surgical Mask 1870+ N95 mask

c. Air+ Protect Mask

d. Other
